# Calcium regulation of HCN supports persistent activity associated with working memory: a multiscale model of prefrontal cortex

**DOI:** 10.1186/1471-2202-15-S1-P108

**Published:** 2014-07-21

**Authors:** Samuel A Neymotin, Robert A McDougal, Michael Hines, William W Lytton

**Affiliations:** 1 Department of Physiology & Pharmacology, SUNY Downstate, Brooklyn, NY, 11203, USA; 2 Department of Neurobiology, Yale Medical School, New Haven, CT, 06510, USA; 3 Department of Neurology, Kings County Hospital Center, Brooklyn, NY, 11203, USA

## 

The Hyperpolarization-activated cyclic-nucleotide gated channel (HCN; *I_h_* current) has been hypothesized to facilitate short-term memory storage in the prefrontal cortex (PFC) via a cascade of signalling events involving second messengers including protein kinases and intracellular calcium (Ca) accumulation [1]. This memory mechanism is effected via alterations in the dynamics of the network, two scales above the underlying molecular level. To determine the factors that enable persistent activity to emerge, we developed a multiscale model (molecular to network level) of PFC that displays the persistent activity associated with working memory.

The network contained 800 cells arranged in 6 neocortical layers. Cell classes included pyramidal cells, and fast-spiking and low-threshold-spiking interneurons. Each neuron contained Na, K, Ca, and HCN channels. Cells were interconnected probabilistically using detailed anatomical data from M1, with AMPA/NMDA, and GABAA synapses. Intracellular Ca was admitted via NMDA and L-type Ca channels. Intracellular signaling components which contributed to persistent activity included: diffusible Ca, diffusible Ca buffering proteins, intracellular endoplasmic reticulum (ER) compartments for sequestering Ca, diffusible inositol triphosphate (IP3), ER IP3 receptors (IP3R) which release Ca from ER upon IP3/Ca binding, sarco/endoplasmic reticulum Ca-ATP-ase pumps (SERCA) which pump cytosolic Ca into the ER, Ca/IP3 degradation reactions. Pyramidal cells had HCN channels regulated by protein kinases bound to intracellular Ca. The model was implemented in parallel NEURON with reaction-diffusion [2]. 18 seconds of simulation time ran over 24 Intel XEON CPUs in ~7 minutes.

We assessed influences between intracellular signaling and network dynamics to isolate sources needed for emergence of short-term memory representations. We provided excitatory stimuli to a subset of pyramidal cells which *represented* the memory. Stimulus-induced depolarization led to Ca influx which increased HCN conductance and excitability of the stimulated population (firing increased from 0.5-2 Hz). Activation of the stimulated population lasted for 5-10 seconds and also increased beta/gamma (~15-35 Hz) oscillations. In contrast, non-stimulated populations were suppressed (firing rate reduction up to 50%) due to higher inhibition caused by the activated cells providing stronger drive to the interneurons. Knocking out specific pathways showed that persistent activity of activated cells was only viable in the presence of Ca influx, Ca regulation of HCN channels, and interactions between excitatory and inhibitory populations, which led to network-derived inhibition activating HCN. Lowering concentrations and binding rates of Ca buffering proteins reduced the network’s ability to represent specific inputs, due to the higher levels of intracellular Ca saturating HCN channels in all pyramidal neurons. This predicts that Ca buffering proteins contribute to regulation of Ca at time-scales relevant for maintaining distinct short-term memory representations. We also used our model to demonstrate *nonsynaptic plasticity* - initial stimuli cause Ca sequestration in the ER of activated neurons, allowing for later, selective modulation of their excitability via metabotropic glutamate receptor activation of IP3Rs, which releases the stored Ca. Our model demonstrates how electro-chemical interactions at the nanoscale may lead to network consequences that produce persistent activity associated with working memory.

## References

[B1] Winograd M Destexhe A Sanchez-Vives MV Hyperpolarization-activated graded persistent activity in the prefrontal cortex PNAS 2008 15 20 7298 7303 10.1073/pnas.0800360105 18474856 PMC2438244

[B2] McDougal RA Hines ML Lytton WW Reaction-diffusion in the NEURON simulator Front Neuroinformatics 2013 15 28 24298253 10.3389/fninf.2013.00028 PMC3828620

